# Construction of a home-based rehabilitation nursing program integrating traditional Chinese and Western medicine for stroke patients based on mobile health

**DOI:** 10.3389/fneur.2025.1670129

**Published:** 2025-10-08

**Authors:** Yan Xu, Zheng Yan, Menghua Ye, Xinrui Huang, Luyi Huang, Min Xu

**Affiliations:** ^1^Department of Breast Medicine, The First Affiliated Hospital of Zhejiang Chinese Medical University (Zhejiang Provincial Hospital of Chinese Medicine), Hangzhou, Zhejiang, China; ^2^The Second Clinical Medical College, Zhejiang Chinese Medical University, Hangzhou, Zhejiang, China; ^3^The School of Nursing, Zhejiang Chinese Medical University, Hangzhou, Zhejiang, China

**Keywords:** mobile health, stroke, integrated traditional Chinese and Western medicine, home-based rehabilitation, evidence summary

## Abstract

**Objective:**

To construct a home-based rehabilitation nursing program integrating traditional Chinese and Western medicine for stroke patients based on mobile health.

**Methods:**

The initial framework of the program was established through evidence-based research. Subsequently, eight experts were invited to participate in an expert meeting to refine and finalize the program.

**Results:**

Following systematic literature retrieval and screening, 30 relevant studies were selected for analysis. Through rigorous evidence extraction, synthesis, group discussion, and evaluation, a preliminary version of the program comprising 6 first-level items, 22 s-level items, and 51 third-level items was formulated. This draft was then reviewed by eight experts with an average authority coefficient of 0.9. After incorporating their feedback, the final program was revised to include 6 first-level items, 24 s-level items, and 51 third-level items.

**Conclusion:**

The home-based rehabilitation nursing program integrating traditional Chinese and Western medicine for stroke patients based on mobile health, demonstrates strong scientific validity and practical feasibility. It can serve as a valuable reference for clinical nursing practice.

## 1 Introduction

Stroke is an acute cerebrovascular event caused by various vascular factors, leading to cerebral artery occlusion or rupture and resulting in corresponding neurological symptoms and signs (1, 2). Epidemiological data indicate that stroke is the second leading cause of death worldwide, with over 11.2 million new cases annually and accounting for more than 5.5 million deaths globally, thereby imposing a significant burden on families and society (3, 4).

Research indicates that 70–80% of stroke survivors experience functional impairments necessitating home-based rehabilitation (5). However, compromised rehabilitation outcomes frequently arise due to patients’ limited understanding of rehabilitation protocols, poor adherence to home-based programs, and insufficient caregiving capacity among family caregivers (6). Consequently, the development of evidence-based home rehabilitation interventions aimed at optimizing patient recovery has emerged as a critical research priority.

At present, Western medical rehabilitation care remains the standard protocol for stroke rehabilitation (7, 8). With the ongoing development of traditional Chinese medicine (TCM), its holistic perspective and emphasis on preventive medicine align closely with the objectives of “disease prevention” and “health promotion” in family-based care (9). This alignment has facilitated the gradual incorporation of TCM rehabilitation strategies into family and community settings, thereby expanding the scope and depth of family-centered care (10, 11). Importantly, TCM rehabilitation principles demonstrate compatibility with international frameworks: its holistic perspective corresponds with the bio-psycho-social disease model outlined in the International Classification of Functioning, Disability and Health (ICF), while the principle of disease prevention aligns with global rehabilitation goals of early intervention and secondary prevention (12, 13). This convergence has further advanced the modernization of TCM.

In the context of medical digitalization, mobile health (mHealth), characterized by its ability to overcome temporal and spatial limitations, has become an essential element of modern remote rehabilitation (14, 15) International guidelines, such as the 2024 American Physical Therapy Association (APTA) clinical practice guideline for tele-Rehabilitation, clearly recognize the value of mHealth in delivering comprehensive, professional, and continuous rehabilitation services (16). These guidelines emphasize the importance of collaborative decision-making between healthcare providers and patients to facilitate the effective adoption of mHealth (16, 17). Meanwhile, this approach enables stroke patients to receive home-based rehabilitation through remote guidance, thereby optimizing the allocation of medical resources and personalizing rehabilitation programs, highlighting its significant potential for application (18).

While numerous studies have investigated home-based rehabilitation for stroke patients, they primarily focus on Western medical approaches (19, 20). The unique value and potential application of TCM nursing in functional recovery remain largely unrecognized and underutilized. Current research inadequately integrates TCM rehabilitation principles with Western practices and lacks a holistic perspective on disease management. To address these deficiencies, this study employs evidence-based research and structured expert meetings to construct a home-based rehabilitation nursing program integrating traditional Chinese and Western medicine for stroke patients based on mHealth. Aims to fulfill the rehabilitation needs of stroke patients while providing an empirical foundation for future home-based interventions.

## 2 Methods

### 2.1 Establishment of a research team

The research team comprises 9 members, including 1 doctoral supervisor and vice president of the hospital, 2 chief physicians, 2 head nurses, and 4 nursing master’s degree students. The primary responsibilities of the nursing master’s degree students are as follows: (1) conducting literature searches, evaluating the quality of relevant studies, extracting and synthesizing evidence; (2) summarizing and organizing the outcomes and opinions reached during expert meeting. Other team members primarily contribute by guiding the research design and participating in the discussion and formulation of the initial draft of the program.

### 2.2 Evidence-based research

#### 2.2.1 Literature search

According to the “6S” model, a comprehensive top-down literature search will be conducted across domestic and international databases and relevant professional association websites, such as the international guidelines network, the website of the China Association of Chinese Medicine, the website of the National Administration of Traditional Chinese Medicine, the JBI EBP Database, the Cochrane Library, CINAHL, PubMed, Embase, Web of Science, CNKI, Wanfang Database, and VIP Database. The search strategy combines subject headings with free-text terms, and is adapted as appropriate according to the specific features of each database. The search period is from the establishment of the database to December 2024. Search terms included: “stroke/cerebral infarction/cerebral hemorrhage/brain vascular accident*/cere-brovascular stroke/cerebrovascular accident*/cerebrovascular apoplexy” “rehabilitation/home rehabilitation/home exercise/home care service/integrative medicine/traditional Chinese medicine nursing/TCM nursing” “guideline/evidence summary/systematic review/meta-analysis/consensus/standard”.

#### 2.2.2 Literature inclusion and exclusion criteria

Inclusion criteria: (1) The study population consisted of stroke patients aged 18 years or older; (2) Study types included guidelines, evidence summaries, systematic reviews, expert consensus statements, and clinical standards; (3) Study content focused on interventions related to home-based rehabilitation and integrated traditional Chinese and Western medicine nursing for stroke patients during the recovery phase. Exclusion criteria: (1) Duplicate publications, outdated versions, or translated versions of guidelines; (2) Research protocols or study plans; (3) Literature with a quality assessment rating of C or lower; (4) Articles published in languages other than Chinese or English; (5) Full-text articles not accessible.

#### 2.2.3 Literature quality evaluation

The guidelines were evaluated using the Appraisal of Guidelines for Research and Evaluation II (AGREE II) (21). The summary of evidence was assessed with the Critical Appraisal for Summaries of Evidence (CASE) (22). The systematic reviews, expert consensus, and standards were evaluated using the corresponding quality appraisal tools from the JBI Evidence-Based Health care Center (23, 24). To ensure scientific rigor in the evaluation process, the assessments were conducted by nursing master’s students who had received training in evidence-based practice. Specifically, the guideline evaluations were carried out by four researchers, and the Intraclass Correlation Coefficient (ICC) was employed to assess inter-rater reliability. The remaining literature was independently evaluated by two researchers. In the event of discrepancies, a third party resolved any disagreements.

#### 2.2.4 Evidence extraction and draft development

Following multiple readings of the included literature by two researchers, evidence was independently extracted and subsequently discussed in-depth with other members of the research team. Based on evidence-based methodologies and consensus reached through team discussions, an initial draft was developed for the home-based rehabilitation nursing program integrating traditional Chinese and Western medicine for stroke patients based on mHealth.

### 2.3 Expert meeting

#### 2.3.1 Expert selection

The inclusion criteria for expert participants were as follows: (1) Professionals in the fields of neurology, rehabilitation medicine, rehabilitation nursing, TCM nursing, or related disciplines, holding a bachelor’s degree or higher, with an associate senior professional title or above, and at least 10 years of clinical work experience; (2) Computer engineers with a master’s degree or higher and a minimum of 5 years of experience in platform and software development; (3) Willingness to actively participate in this study.

#### 2.3.2 Expert consultation form

The Expert Consultation Form is composed of three main sections: (1) Letter to Experts: outlining the research background, objectives, significance, instructions for completing the form, and the researcher’s contact information; (2) Expert Information Survey Form: comprising a basic information survey (covering demographic details such as age, working years, professional title, highest academic qualification, and professional direction) and an authority level assessment form (involving the expert’s self-evaluation of their familiarity with the research topic and the basis for their judgment); (3) Consultation form for the home-based rehabilitation nursing program integrating traditional Chinese and Western medicine for stroke patients based on mHealth: encompassing sections on feasibility evaluation, modification suggestions, and proposed new items.

#### 2.3.3 Implementation process

(1) Pre-Meeting Preparation: Prior to the meeting, the researcher proactively contacts and invites the participating experts, distributes the draft of the program to all experts via email, communicates the research objectives and meeting agenda, confirms the time and location of the meeting, and obtains informed consent from each expert. (2) Conducting the Meeting: At the beginning of the meeting, the researcher briefly introduced the research background, the process of formulating the draft and the research methods, and explained the requirements for filling out the consultation form. This expert meeting adopted a single-round format and lasted approximately 90 min. To ensure the independence of expert opinions as much as possible and reduce group thinking, the following measures were taken: Firstly, the researcher remained neutral when introducing all items and did not express personal preferences. Secondly, before the meeting, experts were invited to fill out the consultation form anonymously and independently, and put forward their initial modification suggestions in writing. In addition, the meeting host paid special attention to ensuring that each expert, especially those with different opinions, had sufficient opportunities to express their views. Subsequently, the meeting entered a real-time item-by-item discussion phase. For each modification suggestion, experts fully explored its rationality and feasibility. Throughout the meeting, two master’s degree students in nursing served as recorders, recording each suggestion and the discussion results in detail in real time. If there were any ambiguities, they would promptly verify and confirm with the experts to ensure the accuracy and completeness of the meeting content. (3) Post-Meeting Procedures: Within 24 h following the meeting, the research team members reviewed the recorded materials based on notes and audio recordings, conducted item-by-item analysis, and finalized the revised draft after internal team discussions and revisions.

#### 2.3.4 Data organization and analysis

Statistical analysis was performed using Excel and SPSS 25.0 software. Descriptive statistics, including frequency and percentage, were used to summarize the general characteristics of the experts. The authority level of the experts was assessed using the authority coefficient (Cr), calculated as follows: Cr = (Ca + Cs)/2. In this formula, Ca represents the coefficient reflecting the basis of experts’ judgments on the research content, which incorporates four dimensions: practical experience, theoretical analysis, reference to domestic and international literature, and intuitive perception. Each dimension is assigned a value based on its influence level, categorized into three grades: high, medium, and low. Cs denotes the coefficient indicating the experts’ familiarity with the research content, classified into five levels. Typically, a Cr value greater than 0.7 suggests acceptable reliability of the consultation outcomes, while a Cr value exceeding 0.8 indicates a high level of reliability (25).

## 3 Results

### 3.1 The results of evidence-based research

Initially, a total of 4,682 publications were retrieved. After repeated screening, 30 literatures were finally included, comprising 12 guidelines (8, 17, 20, 26–34), 1 evidence summary (35), 12 systematic reviews (36–47), 4 expert consensus documents (48–51), and 1 standard (52). A total of 63 pieces of evidence were extracted and synthesized. Based on this synthesis, the research team conducted multiple rounds of discussion and analysis, resulting in a draft consisting of 6 first-level items, 22 s-level items, and 51 third-level items.

### 3.2 The result of the expert meeting

#### 3.2.1 Expert profile

The expert meeting involved a total of 8 participants, with a mean age of 47.50 ± 5.83 years and an average professional experience of 24.13 ± 9.34 years. The panel consisted of 1 expert in neurology, 1 in TCM internal medicine, 1 in rehabilitation medicine, 2 in TCM nursing, 3 in neurology nursing, 1 in nursing informatics, and a software engineer. Among them, two experts are engaged in multiple research disciplines. Detailed demographic and professional information of the experts is presented in Table 1.

**Table 1:** Demographics of the expert meeting.

Characteristics	Number (%)
Sex	
Male	1 (12.5)
Female	7 (87.5)
Age (years)	
<40	1 (12.5)
40–50	4 (50)
>50	3 (37.5)
Working years	
<15	2 (25)
15–30	3 (37.5)
>30	3 (37.5)
Title	
middle level	1 (12.5)
senior level	7 (87.5)
Highest academic qualification	
Undergraduate	1 (12.5)
Master	4 (50)
Doctor	3 (37.5)
Professional direction	
Neurology	1 (10)
TCM internal medicine	1 (10)
Rehabilitation medicine	1 (10)
TCM nursing	2 (20)
Neurology nursing	3 (30)
Nursing informatics	1 (10)
Software engineer	1 (10)

#### 3.2.2 The degree of expert authority

Following the calculation, the Cr of the expert meeting was determined to be 0.9, indicating that the opinions expressed by the experts regarding this plan carry a relatively high level of credibility. The experts’ Ca and Cs scores are shown in Table 2.

**Table 2:** Experts’ Ca and Cs scores.

Number	Ca	Cs
1	1	0.9
2	1	0.9
3	0.9	0.9
4	0.9	0.8
5	0.9	0.9
6	0.9	0.9
7	0.9	0.9
8	0.9	0.8

#### 3.2.3 Expert opinions and revision results

The expert meeting generated a total of 18 recommendations, involving 1 first-level item, 3 s-level items, and 14 third-level items. Following on-site discussion, 17 of these recommendations were ultimately adopted. The summary of key expert suggestions and revisions to the rehabilitation program are shown in Table 3.

**Table 3:** The summary of key expert suggestions and revisions to the rehabilitation program.

Number	Original suggestion	Adoption status	Final revised version
1	The first-level item “Pre-rehabilitation assessment” is not quite appropriate. Home rehabilitation should be subject to full-process assessment. It is suggested to modify it to “Rehabilitation assessment.”	Adopt	2. Rehabilitation assessment
2	“Rehabilitation assessment” should conduct a comprehensive evaluation of the patient from both traditional Chinese and Western medical perspectives. It is suggested that the content related to the “four diagnostic methods of TCM” be supplemented.	Adopt	2.2 Four diagnostic methods of TCM
			2.2.1 Conduct TCM syndrome type differentiation for stroke patients.
3	Cognitive function assessment is an important part of measuring the rehabilitation effect of stroke patients and guiding subsequent treatment. It is recommended to add this assessment content.	Adopt	2.7 Cognitive function
			2.7.1 Mini-Mental State Examination(MMSE)
4	Cognitive function rehabilitation and language function rehabilitation are closely linked. Many rehabilitation activities not only help improve cognitive function but also promote the recovery of language ability. The two can be combined.	Adopt	4.3 Language and cognitive function rehabilitation
5	Members of the multidisciplinary team should cover as many professional fields as possible to meet the diverse needs of patients. It is recommended that on the basis of the existing team, “nutritionists, pharmacists, stroke patients and caregivers” be added.	Adopt	1.1.1 Establish a multidisciplinary home rehabilitation team consisting of doctors, nurses, rehabilitation specialists, psychologists, nutritionists, pharmacists, information technology professionals, stroke patients, caregivers, etc.
6	Simplify the classification of doctors and there is no need to emphasize TCM and Western medicine separately. Just refer to them as doctors.	Adopt	
7	The selection of assessment scales should be based on the actual condition of the patient, and this key point should be emphasized in the “assessment principles.”	Adopt	2.1.1 Based on the patient’s specific condition, standardized and valid assessment tools assessment tools should be selected to conduct a comprehensive evaluation of the patient’s TCM syndrome type, overall health status, functional activity limitations, and home environment. The outcomes of this assessment should be communicated clearly to both the patient and their caregivers in order to facilitate informed decision-making and ensure adequate family support.
8	It is suggested that the relevant content about TCM syndrome type, be supplemented in the “assessment principles.”to reflect the comprehensive assessment method of integrating traditional Chinese and Western medicine.	Adopt	
9	During the home rehabilitation process, the assessment work should be given due attention. It is emphasized that an assessment should be conducted as early as possible before home rehabilitation and regular assessments should be carried out during the rehabilitation period.	Adopt	2.1.2 Conduct assessments as early as possible before home rehabilitation and conduct regular assessments during rehabilitation to guide personalized home rehabilitation plans.
10	The assessment of motor function involves multiple dimensions. It is recommended that the existing scales be systematically classified into three categories: movement, walking, and spasm to ensure the comprehensiveness and specificity of the assessment.	Adopt	2.3.1 (1) Movement: MRC Muscle Strength Scale or Brunnstrom Assessment(BRS); (2)Walking: Holden walking ability rating scale or Wisconsin Gait Scale (WGS) (3); Spasm: Modified Ashworth Scale (MAS), etc.
11	The Fugl-Meyer Assessment (FMA) has a complex assessment content and requires a high level of professional ability from the assessor, making its application in a home setting rather difficult. It is recommended to be removed.	Adopt	
12	When assessing the safety of the home environment for stroke patients, particular attention should be paid to various risk factors that may cause patients to fall. It is recommended to specifically list several areas that may have safety hazards.	Adopt	2.10.1 Assessment of the safety of the patient’s home environment and the need for equipment and home modification, focusing particularly on hazard assessment in stairways, kitchens, and bathrooms. When home visits are infeasible, conduct structured interviews supplemented by photographic/video documentation provided by patients or caregivers.
13	Under the framework of a multidisciplinary team, the participation of patients and their caregivers is a natural component of the home rehabilitation process and does not require additional emphasis. It is recommended that this item be deleted.	Adopt	This item has been deleted.
14	The aerobic exercise section should be supplemented with muscle strength-related Settings.	Adopt	4.1.4 Clinically stable patients with muscle strength ≥ MRC grade 4 should engage in individualized aerobic exercise programs such as walking, table tennis, and Ba Duan Jin.
15	There are certain safety risks in performing moxibustion at home. It is recommended that this item be removed.	Not adopted	5.2.4 Provide “Internet +” homecare services for patients, including individuals with motor dysfunction (e.g., gua sha, moxibustion), dysphagia (e.g., auricular point pressing).
16	Moxibustion techniques can be carried out through “Internet +” homecare services. Meanwhile, it is suggested to list some “Internet +” homecare service items.	Adopt	
17	The implementation of “compensatory strategies to promote swallowing” at home is rather difficult. It is suggested that this be deleted.	Adopt	This item has been deleted.
18	Supplementing acupoints related to cognition in acupoint massage	Adopt	4.3.5 Acupoint massage may facilitate language and cognitive recovery. Apply pressure to GV20 (Baihui), EX-HN5 (Taiyang), and EX-HN1 (Sishencong) for 2–3 min per acupoint, 2–3 times daily.

Finally, based on the recommendations of the expert meeting, the research team revised and refined the initial draft of the program, resulting in the final version of the home-based rehabilitation nursing program integrating traditional Chinese and Western medicine for stroke patients based on mHealth. The final program includes 6 first-level item, 24 s-level items, and 51 third-level items. The specific content is shown in Table 4.

**Table 4:** The home-based rehabilitation nursing program integrating traditional Chinese and Western medicine for stroke patients based on mHealth.

First-level item	Second-level items	Third-level items
1. Organizational management	1.1 Multidisciplinary team	1.1.1 Establish a multidisciplinary home-based rehabilitation team consisting of doctors, nurses, rehabilitation specialists, psychologists, nutritionists, pharmacists, information technology professionals, stroke patients, caregivers, etc.
		1.1.2 All professional members of the rehabilitation team should have specialized training in stroke care and recovery.
	1.2 File management	1.2.1 Collect basic information about stroke patients, including medical history and treatment status, sign informed consent forms for home rehabilitation, and establish medical records.
2. Rehabilitation assessment	2.1 Assessment principles	2.1.1 Based on the patient’s specific condition, standardized and valid assessment tools assessment tools should be selected to conduct a comprehensive evaluation of the patient’s TCM syndrome type, overall health status, functional activity limitations, and home environment. The outcomes of this assessment should be communicated clearly to both the patient and their caregivers in order to facilitate informed decision-making and ensure adequate family support.
		2.1.2 Conduct assessments as early as possible before home rehabilitation and conduct regular assessments during rehabilitation to guide personalized home rehabilitation plans.
	2.2 Four diagnostic methods of TCM	2.2.1 Conduct TCM syndrome type differentiation for stroke patients.
	2.3 Motor function	2.3.1 (1)Movement: MRC Muscle Strength Scale or Brunnstrom Assessment(BRS); (2)Walking: Holden walking ability rating scale or Wisconsin Gait Scale (WGS) (3); Spasm: Modified Ashworth Scale (MAS), etc.
	2.4 Activities of daily living	2.4.1 Barthel Index or FIM
	2.5 Swallowing function	2.5.1 Water swallow test or Standardized Swallowing Assessment (SSA).
	2.6 Language function	2.6.1 Assess speech, language use, reading, and writing through interviews, conversations, observations, standardized tests, or non-standardized projects to identify strengths and weaknesses in communication and determine useful compensatory strategies.
	2.7 Cognitive function	2.7.1 Mini-Mental State Examination(MMSE)
	2.8 Psychological status	2.8.1 Hamilton Rating Scale for Anxiety (HAM-A) or Hamilton Rating Scale for Depression (HAM-D).
	2.9 Fall risk	2.9.1 Berg Balance Scale (BBS) or Morse Fall Scale (MFS).
	2.10 Home environment	2.10.1 Assessment of the safety of the patient’s home environment and the need for equipment and home modification, focusing particularly on hazard assessment in stairways, kitchens, and bathrooms. When home visits are infeasible, conduct structured interviews supplemented by photographic/video documentation provided by patients or caregivers.
	2.11 mHealth literacy	2.11.1 Assess whether stroke patients and their caregivers demonstrate potential to benefit from telehealth technologies (e.g., tablets, web platforms, or communication aids), and adjust the home-based rehabilitation plan accordingly.
3. Implementation of rehabilitation	3.1 Rehabilitation goals and plans	3.1.1 Develop a personalized home rehabilitation plan based on the functional impairments of stroke patients, rehabilitation goals, and social and environmental factors, and review and update it regularly.
	3.2 Timing, frequency, and intensity of rehabilitation	3.2.1 Home-based rehabilitation should begin within 48 h of discharge from an acute hospital or within 72 h of discharge from inpatient rehabilitation. Initial intensity should match hospital-based levels and be progressively titrated based on patient goals and functional outcomes.
		3.2.2 Based on the actual situation of stroke patients, personalized home-based rehabilitation training should be carried out. It is recommended that each type of rehabilitation content last for at least 30–45 min per day, 2–5 days per week, and last for at least 8 weeks.
4. Rehabilitation content	4.1 Motor function rehabilitation	4.1.1 Bedridden patients should maintain proper positioning. Concurrently, they should gradually undergo postural transition training and range-of-motion (ROM) exercises
		4.1.2 Patients with impaired muscle strength should engage in individualized progressive resistance training programs.
		4.1.3 For patients who have difficulty standing or are at risk of falling, activities that challenge balance should be provided.
		4.1.4 Clinically stable patients with muscle strength ≥ MRC grade 4 should engage in individualized aerobic exercise programs such as walking, table tennis, and Ba Duan Jin.
		4.1.5 Deliver structured training in basic activities of daily living, including dressing, feeding, and personal hygiene, to enhance functional independence.
		4.1.6 Recommend a combination of different types of exercise and encourage families and caregivers to participate together.
		4.1.7 Gently pat the Large Intestine Meridian of Hand-Yang-ming and the Stomach Channel of Foot-Yangming twice daily for 30 min each session. This practice may help enhance muscular strength and improve mobility limitations.
		4.1.8 Acupoint massage may improve peripheral circulation and help alleviate symptoms such as limb stiffness and paresthesia. Upper limbs: LI11 (Quchi), LI10 (Shousanli), LI4 (Hegu). Lower limbs: GB30 (Huantiao), BL40 (Weizhong), ST36 (Zusanli). Apply digital pressure to each acupoint for 30–60 s, with a total treatment time of 3–5 min per limb, once daily.
		4.1.9 Apply herbal thermotherapy (38–40 °C) to affected limb sites with pain or edema for 15–30 min per session, once or twice daily.
	4.2 Swallowing function rehabilitation	4.2.1 It is recommended that stroke patients with dysphagia undergo systematic, individualized, and adequate training involving tongue muscle exercises, the Shaker exercise, Masako maneuver,etc.
		4.2.2 Cold stimulation using TCM popsicles or sprays may contribute to the improvement of patients’ swallowing function.
		4.2.3 Acupoint massage at CV23 (Lianquan), GB20 (Fengchi), and TE17 (Yifeng) may help regulate meridian flow and promote swallowing rehabilitation. Apply 2–5 min of pressure per acupoint, 2–3 times daily.
	4.3 Language and cognitive function rehabilitation	4.3.1 In the early stage, targeted interventions can be implemented to address the patient’s impairments in hearing, language expression, reading, writing, and narrative comprehension skills.
		4.3.2 When understanding is difficult, use other means of communication as appropriate, such as gestures, drawing, writing, and assistive and other communication devices.
		4.3.3 It is recommended that patients utilize devices such as computers and virtual reality systems to enhance cognitive and communicative functions.
		4.3.4 Provide patients with opportunities to participate in social and participatory activities, such as conversation partners and community aphasia groups.
		4.3.5 Acupoint massage may facilitate language and cognitive recovery. Apply pressure to GV20 (Baihui), EX-HN5 (Taiyang), and EX-HN1 (Sishencong) for 2–3 min per acupoint, 2–3 times daily.
	4.4 Psychological counseling	4.4.1 For patients with emotional disorders, non-pharmacological interventions should be prioritized. When necessary, these individuals should undergo professional assessment and receive appropriate treatment.
		4.4.2 It is recommended that motivational interviews and personalized education be provided to stroke patients to enhance their proactive participation in rehabilitation and boost their confidence in recovery.
		4.4.3 Recommend that family members, and friends of patients provide them with more companionship and support.
		4.4.4 Encourage patients to actively participate in recreational activities to relieve negative emotions.
5. Remote rehabilitation operation	5.1 Equipment usage training	5.1.1 Patients and their caregivers who receive remote rehabilitation treatment should be trained and supported in the use of the appropriate technology.
	5.2 Remote service management	5.2.1 Remote service technologies and processes should be implemented to ensure the timely and accurate recording of patient information.
		5.2.2 Ensure the effectiveness, privacy and reliability of the equipment.
		5.2.3 Regularly inspect the equipment to ensure it functions properly in emergency situations.
		5.2.4Provide “Internet +” homecare services for patients, including individuals with motor dysfunction (e.g., gua sha, moxibustion), dysphagia (e.g., auricular point pressing).
	5.3 Monitoring feedback	5.3.1It is recommended that a professional be assigned to supervise the implementation of the home rehabilitation plan.
		5.3.2 Wearable devices are used to monitor patients’ vital signs during home rehabilitation, and alerts are issued for abnormal values to ensure the safety of home rehabilitation.
		5.3.3 It is recommended to use a rehabilitation log or set up rehabilitation reminders to track the progress of stroke patients’ home rehabilitation.
		5.3.4 Communicate with patients online, with designated personnel answering patient questions on a regular basis, while also advising patients to seek medical attention promptly if they experience any adverse symptoms.
		5.3.5 It is recommended that stroke patients provide regular feedback on their rehabilitation progress and outcomes, for example, by using recorded audio or video.
6. Health education	6.1 Educational formats	6.1.1 It is recommended to use a variety of formats, such as graphic materials, videos, and audio recordings.
	6.2 Educational content	6.2.1 Ensure that health education content is comprehensive, including guidance on disease knowledge, medication, diet, stroke recurrence identification, and home safety education.

## 4 Discussion

### 4.1 The scientificity and reliability of the process of formulating the plan

This study was conducted using evidence-based research methodology. A comprehensive literature search was carried out across both Chinese and English databases, as well as on the official websites of major professional associations. The retrieved literature was rigorously screened and synthesized to ensure the scientific validity and reliability of the evidence sources. Based on the findings, the research team obtained the draft of the program, which was further discussed in an expert meeting. Through face-to-face communication and collaborative discussion, each item was carefully revised to better align with the current clinical practices in China. The experts participating in the meeting are highly accomplished professionals with extensive clinical experience and academic expertise in relevant fields such as clinical medicine, nursing, rehabilitation, and information technology. Their multidisciplinary perspectives contributed valuable insights and further enhanced the scientific rigor of the program. Among the seven medical experts involved in the meeting, six hold a master’s degree or higher, and all hold associate senior-level professional titles or above, indicating strong representativeness. The Cr is 0.9, reflecting a high level of expertise and confidence in the outcomes of the discussion, thereby ensuring the reliability and credibility of the final program.

### 4.2 The comprehensiveness and practicality of the plan content

#### 4.2.1 A well-structured management system ensures the continuity of the plan

This study clarifies the role composition of the multidisciplinary team and integrates multidisciplinary resources to provide systematic, comprehensive and professional continuous care services for stroke patients. On this basis, the program emphasizes that “all professional members of the rehabilitation team should have specialized training in stroke care and recovery,” aiming to enhance the overall professional quality and nursing service level of the team (26). At the same time, a complete medical record effectively solves the problems of untimely information tracking and fragmented records in traditional follow-up, providing necessary information support for medical staff to fully understand the patient’s condition, formulate and dynamically adjust individualized home-based rehabilitation plans, and further ensure the continuity and effectiveness of home-based rehabilitation (53).

#### 4.2.2 Assessment serves as the prerequisite and foundation for home-based rehabilitation

This program adheres to established assessment principles and conducts a systematic evaluation of patients. Building upon this, it integrates the four diagnostic methods of TCM with conventional Western medical assessment techniques, thereby establishing a multi-dimensional and standardized rehabilitation assessment framework that reflects the integration of traditional Chinese and Western medicine. Moreover, the program specifies applicable assessment tools, facilitating implementation by multidisciplinary teams. Given that the elderly constitute the primary population of stroke patients, who often experience limb dysfunction and declining physical abilities, their acceptance of mobile healthcare remains relatively low (5, 54). Therefore, a comprehensive assessment of patients’ mHealth literacy is essential to ensure the program’s applicability and effectiveness. Additionally, during the evaluation process, special attention should be paid to potential difficulties that patients may encounter when operating mobile devices, such as complex interfaces and unclear fonts. The results from these evaluations can provide a basis for universal improvements in mHealth devices and inform targeted training initiatives, thereby enhancing both the overall feasibility and user experience of the rehabilitation program.

#### 4.2.3 Optimize the rehabilitation content and enhance the comprehensiveness of the program

This program clearly outlines the goals and objectives of home-based rehabilitation, with a focus on the timing, frequency, and intensity of such interventions. Medical personnel are empowered to make dynamic adjustments based on ongoing rehabilitation assessments and in consideration of each patient’s individual circumstances. In terms of content, the program addresses four key domains: motor function, swallowing, language and cognitive abilities, and psychological well-being. The rehabilitation framework is comprehensive, detailed, and highly practical, offering structured guidance for healthcare providers, patients, and other relevant stakeholders. Furthermore, as public health awareness continues to grow and TCM receives increasing governmental support and promotion, there is a rising demand for TCM nursing services among the general population (55). In response to this need, the program integrates evidence-based and operationally feasible TCM nursing techniques into conventional rehabilitation practices. Detailed instructions regarding implementation methods and recommended frequencies are provided, with the ultimate goal of delivering holistic and integrated rehabilitation support that facilitates comprehensive functional recovery. It is worth noting that the different reserves of traditional Chinese medicine knowledge among patients may also affect the effective implementation of home-based traditional Chinese medicine techniques. Therefore, it is recommended that, based on the actual situation, training on traditional Chinese medicine care-related knowledge be provided to patients and their caregivers to enhance the patients’ self-management ability.

#### 4.2.4 Strengthen remote rehabilitation support and enhance the feasibility of the program

As an emerging model of home-based rehabilitation, current mHealth services for stroke patients require further refinement in terms of specific intervention formats and procedural frameworks (56). To address this gap, the program seeks to enhance patient user experience through systematic equipment training and comprehensive technical support. Additionally, it aims to effectively mitigate challenges such as the lack of supervision during the home-based rehabilitation process by implementing regular monitoring and timely feedback mechanisms, thereby improving the overall practicality and feasibility of the program. Furthermore, taking into account the needs of patients with limited mobility or those requiring immediate medical guidance, and acknowledging that although traditional Chinese medical techniques such as moxibustion and gua sha offer notable therapeutic benefits, they also present certain operational challenges and safety risks when performed at home, this program innovatively incorporates “Internet +” homecare services. This approach not only extends the scope and depth of nursing care but also ensures the safety and efficacy of home-based rehabilitation interventions (57). However, continuous efforts are still needed to address potential issues such as patients’ compliance with home rehabilitation and the related risks brought about by operating without on-site supervision by professionals.

## 5 Limitations

The following are the limitations of this study. First, the sample was limited to experts from Zhejiang Province; future research should include participants from diverse geographical regions in order to enhance the generalizability of the findings. Second, due to time constraints, randomized controlled trials have not yet been conducted to evaluate the effectiveness of the intervention. Nevertheless, the research team has developed a mHealth platform based on the program and intends to carry out further intervention studies to assess the long-term efficacy of the approach.

## 6 Conclusion

This study utilized evidence-based research and expert meeting to develop a home-based rehabilitation nursing program integrating traditional Chinese and Western medicine for stroke patients based on mHealth. The program demonstrates scientific validity and clinical applicability, and is capable of addressing the diverse needs of patients. However, it is recommended that the implementation of the program be adapted to the actual medical resource conditions in different regions.

## Data Availability

The original contributions presented in the study are included in the article/supplementary material, further inquiries can be directed to the corresponding author/s.

## References

[1] Tu WJ Wang LD. China stroke surveillance report 2021. Mil Med Res. (2023) 10:33. doi: 10.1186/s40779-023-00463-x, PMID: 37468952 PMC10355019

[2] Feigin VL Brainin M Norrving B Martins SO Pandian J Lindsay P. World stroke organization: global stroke fact sheet 2025. Int J Stroke. (2025) 20:132–44. doi: 10.1177/17474930241308142, PMID: 39635884 PMC11786524

[3] Feigin VL Brainin M Norrving B Martins S Sacco RL Hacke W. World stroke organization (WSO): global stroke fact sheet 2022. Int J Stroke. (2022) 17:18–29. doi: 10.1177/17474930211065917, PMID: 34986727

[4] Wu S Wu B Liu M Chen Z Wang W Anderson CS. Stroke in China: advances and challenges in epidemiology, prevention, and management. Lancet Neurol. (2019) 18:394–405. doi: 10.1016/S1474-4422(18)30500-3, PMID: 30878104

[5] MacKay-Lyons M Billinger SA Eng JJ Dromerick A Giacomantonio N Hafer-Macko C. Aerobic exercise recommendations to optimize best practices in care after stroke: AEROBICS 2019 update. Phys Ther. (2020) 100:149–56. doi: 10.1093/ptj/pzz153, PMID: 31596465 PMC8204880

[6] Zeng S Wu M Xu L Guo Z Chen S Ling K. Challenges in accessing community-based rehabilitation and long-term care for older adult stroke survivors and their caregivers: a qualitative study. J Multidiscip Healthc. (2024) 17:4829–338. doi: 10.2147/JMDH.S476993 39464787 PMC11512762

[7] Gittler M Davis AM. Guidelines for adult stroke rehabilitation and recovery. JAMA. (2018) 319:820–1. doi: 10.1001/jama.2017.22036, PMID: 29486016

[8] Hebert D Lindsay MP McIntyre A Kirton A Rumney PG Bagg S. Canadian stroke best practice recommendations: stroke rehabilitation practice guidelines, update 2015. Int J Stroke. (2016) 11:459–84. doi: 10.1177/1747493016643553, PMID: 27079654

[9] Gao Y. Construction of a comprehensive diagnosis and treatment plan for stroke diseases and patterns based on the holistic view of traditional Chinese medicine. J Beijing Univ Tradit Chin Med. (2024) 47:4–8. doi: 10.3969/j.issn.1006-2157.2024.01.002

[10] Jin J Zhong MR Yu XT Yu XT Yang TT Fan KY. Construction and application of traditional Chinese medicine continuous nursing program for patients with acute ischemic stroke. Chin J Nurs. (2021) 56:1125–32. doi: 10.3761/j.issn.0254-1769.2021.08.001

[11] Zhu RR Wang JX Pan B Lai HH Xu XT Ge L. Evidence-based evaluation for stroke guidelines mentioning traditional and complementary medicine rehabilitation. BMC Complement Med Ther. (2025) 25:184. doi: 10.1186/s12906-025-04916-9, PMID: 40390019 PMC12090438

[12] Seger W. The rediscovery of the social side of medicine: philosophy and value of the international classification of functioning, disability and health (ICF). Electron Physician. (2018) 10:6426–9. doi: 10.19082/6426, PMID: 29765565 PMC5942561

[13] Li J Zhao X Zhang Y Wan H He Y Li X. Comparison of traditional Chinese medicine in the long-term secondary prevention for patients with ischemic stroke: a systematical analysis. Front Pharmacol. (2021) 12:722975. doi: 10.3389/fphar.2021.722975, PMID: 34867326 PMC8637749

[14] Niyomyart A Ruksakulpiwat S Benjasirisan C Phianhasin L Nigussie K Thorngthip S. Current status of barriers to mHealth access among patients with stroke and steps toward the digital health era: systematic review. JMIR Mhealth Uhealth. (2024) 12:e54511. doi: 10.2196/54511, PMID: 39173152 PMC11377914

[15] Amuasi J Agbogbatey MK Sarfo FS Beyuo A Duah K Agasiya P. Feasibility, acceptability, and appropriateness of a mobile health stroke intervention among Ghanaian health workers. J Neurol Sci. (2022) 439:120304. doi: 10.1016/j.jns.2022.120304, PMID: 35689867

[16] Holland AE. Appraisal of clinical practice guideline: telerehabilitation in physical therapist practice: a clinical practice guideline from the American Physical Therapy Association. J Physiother. (2024) 70:317. doi: 10.1016/j.jphys.2024.07.006 39327182

[17] Blacquiere D Lindsay MP Foley N Taralson C Alcock S Balg C. Canadian stroke best practice recommendations: telestroke best practice guidelines update 2017. Int J Stroke. (2017) 12:886–95. doi: 10.1177/1747493017706239 28441928

[18] Chae SH Kim Y Lee KS Park HS. Development and clinical evaluation of a web-based upper limb home rehabilitation system using a smartwatch and machine learning model for chronic stroke survivors: prospective comparative study. JMIR Mhealth Uhealth. (2020) 8:e17216. doi: 10.2196/17216, PMID: 32480361 PMC7380903

[19] Aphiphaksakul P Siriphorn A. Home-based exercise using balance disc and smartphone inclinometer application improves balance and activity of daily living in individuals with stroke: a randomized controlled trial. PLoS One. (2022) 17:e0277870. doi: 10.1371/journal.pone.0277870, PMID: 36409753 PMC9678269

[20] Winstein CJ Stein J Arena R Bates B Cherney LR Cramer SC. Guidelines for adult stroke rehabilitation and recovery: a guideline for healthcare professionals from the American Heart Association/American Stroke Association. Stroke. (2016) 47:e98–e169. doi: 10.1161/str.0000000000000098, PMID: 27145936

[21] Brouwers MC Kerkvliet K Spithoff K AGREE Next Steps Consortium. The AGREE reporting checklist: a tool to improve reporting of clinical practice guidelines. BMJ. (2016) 354:i4852. doi: 10.1136/bmj.i4852 26957104 PMC5118873

[22] Foster MJ Shurtz S. Making the critical appraisal for summaries of evidence (CASE) for evidence-based medicine (EBM): critical appraisal of summaries of evidence. J Med Libr Assoc. (2013) 101:192–8. doi: 10.3163/1536-5050.101.3.008, PMID: 23930089 PMC3738079

[23] Gu Y Zhang HW Zhou YF Hu Y Xing WJ. JBI evidence-based health care center quality appraisal tools for different types of studies: methodological quality assessment of systematic reviews. J Nurses Train. (2018) 33:701–3. doi: 10.16821/j.cnki.hsjx.2018.08.008

[24] Joanna Briggs Institute. *Critical appraisal tools checklist for text and opinion*. (2017). Available online at: https://jbi.global/critical-appraisal-tools (Accessed Jun 13, 2025).

[25] Guo Y Yang S Boonyamalik P Powwattana A Zhu W Xu L. Development of a social learning theory-based pressure injury training program for nursing assistants in Chinese nursing homes. Front Public Health. (2024) 12:1478147. doi: 10.3389/fpubh.2024.1478147, PMID: 39989863 PMC11842331

[26] Teasell R Salbach NM Foley N Mountain A Cameron JI Jong A. Canadian stroke best practice recommendations: rehabilitation, recovery, and community participation following stroke. Part one: rehabilitation and recovery following stroke; 6th edition update 2019. Int J Stroke. (2020) 15:763–88. doi: 10.1177/1747493019897843, PMID: 31983296

[27] National Health Commission of the People's Republic of China.*Chinese stroke prevention and treatment guidelines*. (2021). Available online at: https://wjw.xinjiang.gov.cn/hfpc/ylzl/202109/c9708dfb6a7f47dd95ec879432f8f1bb.shtml. (Accessed Jun 13, 2025).

[28] Cameron JI O’Connell C Foley N Salter K Booth R Boyle R. Canadian stroke best practice recommendations: managing transitions of care following stroke, guidelines update 2016. Int J Stroke. (2016) 11:807–22. doi: 10.1177/1747493016660102, PMID: 27443991

[29] Scottish Intercollegiate Guidelines Network. *National clinical guideline for stroke for the UK and Ireland*. (2023). Available online at: https://www.strokeguideline.org (Accessed Jun 13, 2025).

[30] National Institute for Health and Care Excellence. *Stroke rehabilitation in adults*. (2019). Available online at: https://www.nice.org.uk/guidance/cg162 (Accessed Jun 15, 2025).

[31] National Stroke Foundation. *Living clinical guidelines for stroke management*. (2022). Available online at: https://informme.org.au/en/Guidelines/Clinical-Guidelines-for-Stroke-Management (Accessed Jun 15, 2025).

[32] Inness E Brown G Tee A Kelly L Moller J Aravind G. The Canadian stroke community-based exercise recommendations update 2020: a resource for community-based exercise providers. Int J Stroke. (2021) 16:60. doi: 10.1177/17474930211041949

[33] Zhang T Zhao J Li XP Bai YL Wang BJ Qu Y. Chinese clinical management guidelines for cerebrovascular diseases (edition 2) (excerpt) - chapter 8 rehabilitation management of cerebrovascular diseases. Chin J Stroke. (2023) 8:1036–48. doi: 10.3969/j.issn.1673-5765.2023.09.010

[34] Ni XJ Chen YL Cai YF. Evidence-based practice guideline for integrated traditional Chinese and Western medicine in stroke (2019). Chin J Evid Based Med. (2020) 20:901–12. doi: 10.7507/1672-2531.202001075

[35] Li XJ. *Optimization study of traditional Chinese medicine nursing protocol for stroke based on evidence body*. The Beijing University of Chinese Medicine (2019).

[36] Xu B Xu JL Yang Y Geng GL. Impact of domestic home nursing on health status of stroke patients: a meta-analysis. Chin J Rehabil Med. (2016) 31:901–12. doi: 10.3969/j.issn.1001-1242.2016.10.014

[37] Chi NF Huang YC Chiu HY Chang HJ Huang HC. Systematic review and meta-analysis of home-based rehabilitation on improving physical function among home-dwelling patients with a stroke. Arch Phys Med Rehabil. (2020) 101:359–73. doi: 10.1016/j.apmr.2019.10.181, PMID: 31689417

[38] Lu R Lloyd-Randolfi D Jones H Connor LT AlHeresh R. Assessing adherence to physical activity programs post-stroke at home: a systematic review of randomized controlled trials. Top Stroke Rehabil. (2021) 28:207–18. doi: 10.1080/10749357.2020.1803573 32787644

[39] Zhang Q Schwade M Smith Y Wood R Young L. Exercise-based interventions for post-stroke social participation: a systematic review and network meta-analysis. Int J Nurs Stud:103738. doi: 10.1016/j.ijnurstu.2020.1037.38 32858433

[40] Nascimento LR Rocha RJS Boening A Ferreira GP Perovano MC. Home-based exercises are as effective as equivalent doses of Centre-based exercises for improving walking speed and balance after stroke: a systematic review. J Physiother. (2022) 68:174–81. doi: 10.1016/j.jphys.2022.05.018, PMID: 35753966

[41] Laver KE Adey-Wakeling Z Crotty M Lannin NA George S Sherrington C. Telerehabilitation services for stroke. Cochrane Database Syst Rev. (2020) 1:CD010255. doi: 10.1002/14651858.CD010255.pub3, PMID: 32002991 PMC6992923

[42] Bok SK Song Y Lim A Jin S Kim N Ko G. High-tech home-based rehabilitation after stroke: a systematic review and meta-analysis. J Clin Med. (2023) 12:12. doi: 10.3390/jcm12072668, PMID: 37048751 PMC10095213

[43] Szeto SG Wan H Alavinia M Dukelow S MacNeill H. Effect of mobile application types on stroke rehabilitation: a systematic review. J Neuroeng Rehabil. (2023) 20:12. doi: 10.1186/s12984-023-01124-9, PMID: 36694257 PMC9872745

[44] Zhang XY Wang P Yan LJ Wang YH Wang D Qu C. Effects of home-based telerehabilitation in stroke survivors: a systematic review. Chin J Evid Based Med. (2019) 19:1226–32. doi: 10.7507/1672-2531.201904004

[45] Ji QL Guan FG. Effects of moxibustion on limb motor function in post-stroke hemiplegic patients: a meta-analysis. Chin J Med Sci. (2020) 10:28–35. doi: 10.3969/j.issn.2095-0616.2020.07.007

[46] Wang JL Dai XJ Shi Q Wu XC. Efficacy of acupoint massage for post-stroke dysphagia: a meta-analysis. Chin Nurs Res. (2020) 34:901–8. doi: 10.12102/j.issn.1009-6493.2020.05.020

[47] He XY Ma QQ Zhai YK Cui FF Lu YE Chen HT. Impact of telerehabilitation on functional recovery in stroke patients: a meta-analysis. Chin J Rehabil Med. (2020) 35:1466–71. doi: 10.3969/j.issn.1001-1242.2020.12.011

[48] Yan JT Yang PJ Wu Y Chen WH Shan CL Hu J. Shanghai expert consensus on home-based stroke rehabilitation. J Shanghai Univ Tradit Chin Med. (2020) 34:10–5. doi: 10.16306/j.1008-861x.2020.02.001

[49] Chinese expert consensus on rehabilitation nursing and care for community-dwelling patients with dysphagia. Chin J Geriatr Health Med. (2019) 17:7–15. doi: 10.3969/j.issn.1672-2671.2019.04003

[50] Mahmood A Deshmukh A Natarajan M Marsden D Vyslysel G Padickaparambil S. Development of strategies to support home-based exercise adherence after stroke: a delphi consensus. BMJ Open. (2022) 12:e055946. doi: 10.1136/bmjopen-2021-055946, PMID: 34992120 PMC8739434

[51] Salbach NM Mountain A Lindsay MP Blacquiere D McGuff R Foley N. Canadian stroke best practice recommendations virtual stroke rehabilitation interim consensus statement 2022. Am J Phys Med Rehabil. (2022) 101:1076–82. doi: 10.1097/phm.0000000000002062, PMID: 35767008

[52] National administration of traditional Chinese medicine. *Traditional Chinese medicine nursing program for stroke and 12 other diseases (trial)*. (2013). Available online at: http://yzs.satcm.gov.cn/gongzuodongtai/2018-03-24/2800.html (Accessed Jun 15, 2025).

[53] Chen X Gong E Tan J Turner EL Gallis JA Sun S. Long-term mortality outcome of a primary care-based mobile health intervention for stroke management: six-year follow-up of a cluster-randomized controlled trial. PLoS Med. (2025) 22:e1004564. doi: 10.1371/journal.pmed.1004564, PMID: 40146698 PMC11949329

[54] Tyagi S Lim DSY Ho WHH Koh YQ Cai V Koh GCH. Acceptance of tele-rehabilitation by stroke patients: perceived barriers and facilitators. Arch Phys Med Rehabil. (2018) 99:2472–2477.e2. doi: 10.1016/j.apmr.2018.04.033, PMID: 29902469

[55] Tang W Tao QY. Clinical follow-up and analysis of community demand and mastery of traditional Chinese medicine appropriate techniques in ischemic stroke patients. Chin Gen Pract. (2019) 22:2495–8. doi: 10.12114/j.issn.10079572.2019.00.185

[56] Xu Y Huang XR Xu M. Application of mobile health in home-based stroke rehabilitation: a scoping review. Chin J Nurs Sci. (2023) 38:121–5. doi: 10.3870/j.issn.1001-4152.2023.17.121

[57] Hu L Chen H Mo W Han Y Sun H. Best evidence summary for management of older people with type 2 diabetes mellitus using 'internet plus nursing servic es'. Int J Older People Nursing. (2024) 19:e12657. doi: 10.1111/opn.12657, PMID: 39429159

